# Effect of climate change on outdoor thermal comfort in humid climates

**DOI:** 10.1186/2052-336X-12-46

**Published:** 2014-02-11

**Authors:** José A Orosa, Ángel M Costa, Ángel Rodríguez-Fernández, Gholamreza Roshan

**Affiliations:** 1 Department of Energy and M. P., University of A Coruña. ETSNyM, Paseo de Ronda 51, 15011 A Coruña, Spain; 2 Department of Geography, Golestan University, Gorgan, Iran

**Keywords:** Climate change, Thermal comfort, Outdoor, Humid

## Abstract

**Background:**

Galicia, in northwest Spain, experiences warm summers and winters. However, the higher relative humidity that prevails the whole year through and the location of the summer hot points are related to real weather heat stroke in the hottest season. However, Planet Global Heating was recently analyzed for the climate in Galicia. Climate change was found to be able to trigger effects that involve a new situation with new potential regions of risk. In this paper, 50 weather stations were selected to sample the weather conditions in this humid region, over the last 10 years. From these results, new regions with a potential for heat stroke risk in the next 20 years were identified using the humidex index.

**Results:**

Results reveal that during the last 10 years, the winter season presents more comfortable conditions, whereas the summer season presents the highest humidex value. Further, the higher relative humidity throughout the whole year reveals that the humidex index clearly depends upon the outdoor temperature.

**Conclusions:**

Global Planet Heating shows a definite effect on the outdoor comfort conditions reaching unbearable degrees in the really hottest zones. Therefore, this effect will clearly influence tourism and risk prevention strategies in these areas.

## Background

European weather changes commence from the Atlantic, and thereafter, the events that occur there determine, to a large degree, the weather and the climate of Europe. Galicia, located in the northwest of Spain enjoys mild climate, mainly influenced by the Azores anticyclone, Iceland depression, and central Europe thermal anticyclone. The Azores anticyclone controls the movement of the depression zones towards the Galician coast, which is influenced by the annual revolution of the Earth around the sun.

However, it is noteworthy that the extreme climatic conditions during the summer months pose a significant public health threat. For example, the unfavourable weather conditions were related [1] to the daily mortality rates during the 30-year period between1968 and 1997, in the moderate climate of SW Germany. From the results recorded between the end of June and the beginning of July, about 25% of the variance in the deviations of the mortality rate from the smoothed values can be attributed to the effects of the thermal environment. The increased mortality, particularly during the heat waves, was more pronounced although it was followed by lower than average values during the subsequent weeks. Other recent research studies on the relationship between mortality and discomfort caused by climatic conditions as well as the short lag time proclaimed a clear public health message: preventive, social and health care actions must be administered to the elderly and the frail to reduce the mortality rate during the period of the heat waves [2].

An important, and most often neglected, parameter in the relationship between weather and mortality is climate change. In this sense, the recent papers [3-6] showed how climate change could alter actual situations. Climate is defined as the average of the climatologic variables over a time span of about 30 years. This effect is assumed to be related to theories based on an astronomic perspective such as the sun’s brightness and earth’s orbit, while others are presented from a geological perspective like continental drift and volcanism; still others are related to the perspective of greenhouse gases and weather patterns like the location of ice caps, the role of the sea currents and blocking the anticyclones [7]. The greenhouse effect, in particular, related to these released gases, results in raising the global planet temperature between 1.4 and 5.8°C, as well as producing a likely substantial increase in the extreme meteorological weather conditions, such as a rise in the sea level between 9 and 88 cm.

Once the possible reasons for global climatic changes happen and their consequences are explained, their effects on future tourism [8] and subsequently the measures necessary to prevent heat stroke in the population can be considered. Further, the chief aim of these models is to prevent heat-related illness and deaths, even as some European cities had developed hot-weather response plans. In this context, in some recent studies [9], the vulnerabilities related to tourism-based activities and locations were referred to generally. Coastal areas, particularly, appear to necessitate careful attention, given their susceptibility to the fluctuating water levels and their significance in tourism and recreation [9]. The same researchers proposed a man-environment heat exchange model (MENEX) [10]. MENEX can be assessed in various applications: bioclimatic (i.e. related to recreation and tourism, climatotherapy, human health, urban studies), thermophysiological (work conditions, controlling of thermoregulation system etc.), spatial design of landscapes, residential and recreational areas. All the components of the MENEX_2005 model and output variables can be calculated using the BioKlima software package which is currently employed in bioclimatic and thermophysiological research in different countries [10].

However, the experimental inductive (field research of micro-climatic variables and subjective answers) and deductive (simulation of predictive models) as in [11,12] methods are other possible options. These studies usually employ a different index based on the actual weather conditions. In this kind of research, as the next logical step, this paper discusses the real effect of climate change on heat stress in the most humid region of Spain, and the analysis will be based on the humidex, one of the most frequently employed indices.

## Methods

### Climatic data

**Figure 1:**
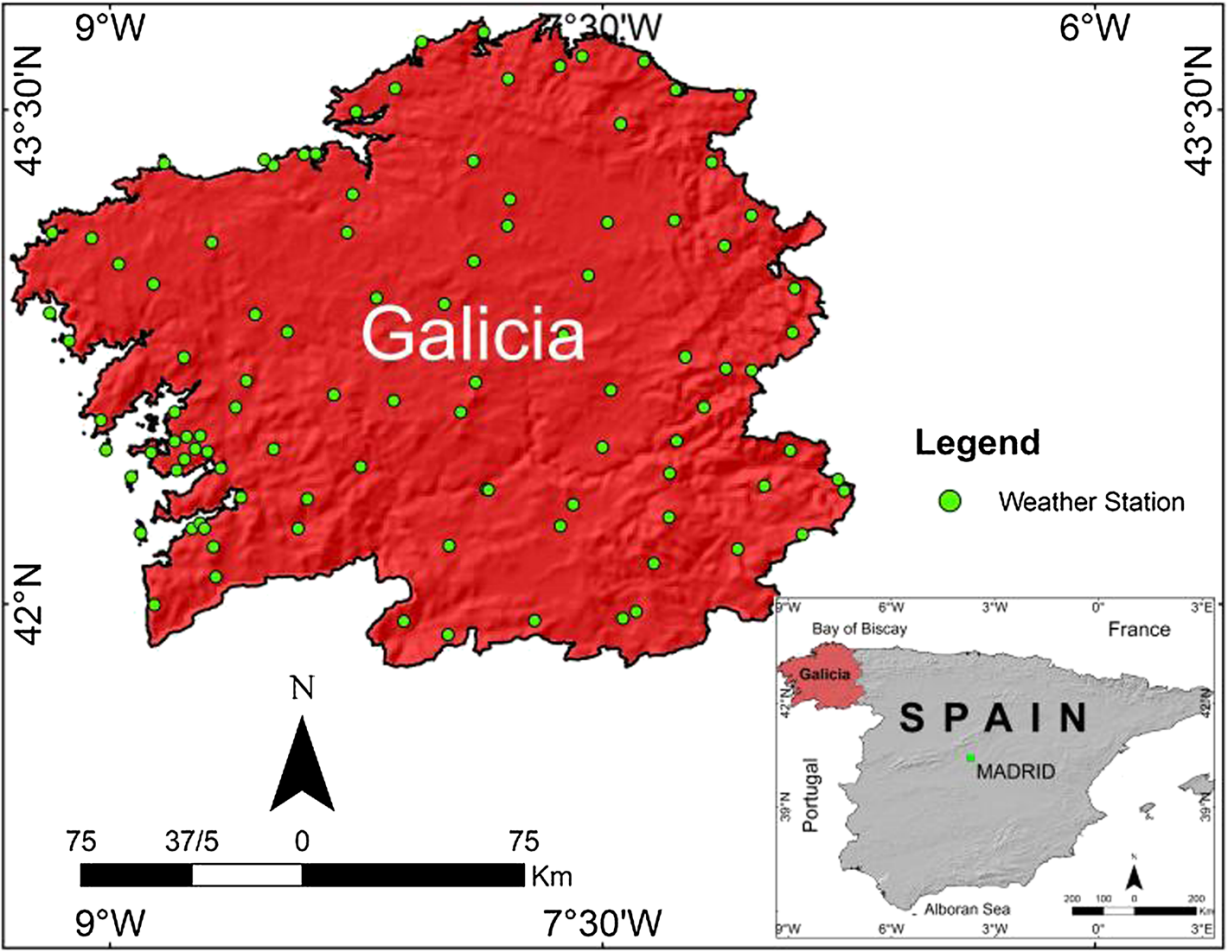
Weather stations.

Climatic data was sampled in 50 weather stations located in the important places in Galicia, as indicated in Figure 1 indicated as white points. Climate there was affected by the Atlantic Ocean winds, implying high relative humidity throughout the year [13]. These meteorological stations show recorded variables such as temperature, relative humidity and wind speed, among others, with a sample frequency of about 5–10 minutes. These weather stations were specifically selected for this study as they avoid buildings and other parameters that could interfere with the sample data, according to the ASHRAE 2005 [14] measuring conditions, as well as their temperature and relative humidity with an error of ±0.1°C and 1%, respectively.

### Climatic change predictions

Climatic change models were obtained from the references [3] after a curve fit was done for each season, also taking into account the linear evolution of temperature increase with the years, according to the Equations 1, 2, 3 and 4 for the winter, spring, summer and autumn seasons, respectively. Each equation shows the average temperature rise in Galicia over the last 30 years, with an average value of 13.63°C in 1987.

(1) $$\Delta t_{\mathrm{winter}}=0.05\cdot \tau$$

(2) $$\Delta t_{\mathrm{spring}}=0.07\cdot \tau$$

(3) $$\Delta t_{\mathrm{Summer}}=0.05\cdot \tau$$

(4) $$\Delta t_{\mathrm{Autumn}}=0.03\cdot \tau$$

Where,

∆*t* is the mean seasonal temperature increment (°C)

*τ* is the time (years)

Once these models are obtained, they can be used to predict the climatic changes in Galicia for the next few years. The year 2030 was chosen due to the great interest in the effect of climate change on the occupation of different parts of Galicia in order to develop future action plans, like civil constructions and architectural building designs, for population protection. From the perspective of preventing heat-related illnesses and deaths, some European cities developed hot-weather response plans [15].

### The humidex index

In some recent research works, two different heat indices were compared; the first index being the National Weather Service (NWS) Heat Index (HI) derived from previous databases and the second being humidex, a Canadian innovation. This index is employed by the Meteorological Services Canadian (MSC) [16]. In 1979, Masterton & Richardson [17] proposed humidex as an index that is calculated, based on air temperature and humidity, devised by the Canadian meteorologists, to describe the experiences of an average person during hot, humid weather [18]. Humidex thus combines temperature and humidity into one number to reflect the temperature perceived, and consequently, a measure of thermal discomfort. As it considers the two most important factors that affect summer comfort, it is a better method of measuring how stifling the air feels, more than either temperature or humidity alone.

An extremely high humidex reading is defined as one that crosses 40, as shown in Table 1. Under such conditions, all unnecessary activity should be curtailed. If the reading lies between the mid and the high 30s, then certain types of outdoor exercise should be toned down or modified, depending on the age and health of the individual, the physical shape, the type of clothes worn and other weather conditions. If working outdoors is an absolute necessity, the intake of plenty of liquids must be mandatory and frequent rest breaks are necessary. Hot, humid conditions precipitate a considerable risk of heat stroke and sun stroke.

**Table 1:** Guide to summer comfort for the different humidex ranges

**Range of humidex: Degree of comfort**	
Less than 29	No discomfort
30 to 39	Some discomfort
40 to 45	Great discomfort; avoid exertion
Above 45	Dangerous
Above 54	Heat stroke imminent

Despite its limitations, humidex remains a useful, and hence popular, means of determining how hot it actually feels outside. Both indices present advantages; for example, the HI presents a linear relationship dependency with the predicted rectal temperature. Humidex thus presents an exponential model which, despite its greater complexity, shows a better average correlation coefficient (R^2^) higher than HI, as we can see in Equation 5.

(5) $$H=T+\left(0.5555\cdot \left(e-10\right)\right)$$

Where T is the temperature in Celsius and e is the vapor pressure in millibars (mb).

Therefore, and in accordance with the Meteorological Services Canada (MSC), humidex was selected.

## Results and discussion

Figures 2, 3, 4, 5, 6, 7, 8, 9, 10 and 11 show the main results obtained. In particular, Figures 2 and 3 show the monthly weather conditions and Figures 4 and 5 show the humidex model. Finally, Figures 6 and 7 shows the instantaneous conditions and Figures 8, 9, 10 and 11 show the expected future conditions.

**Figure 2:**
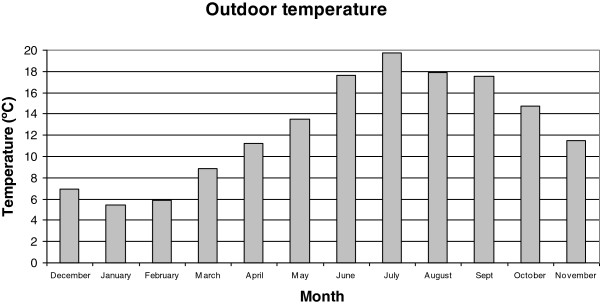
Monthly outdoor temperature in 2008.

**Figure 3:**
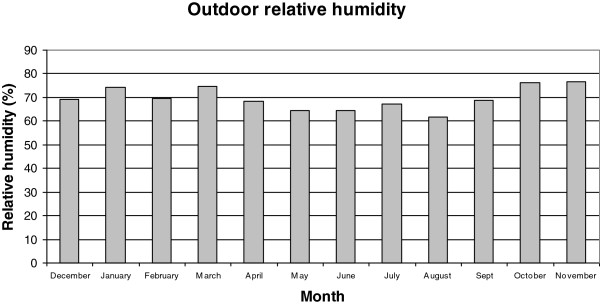
Monthly outdoor relative humidity in 2008.

**Figure 4:**
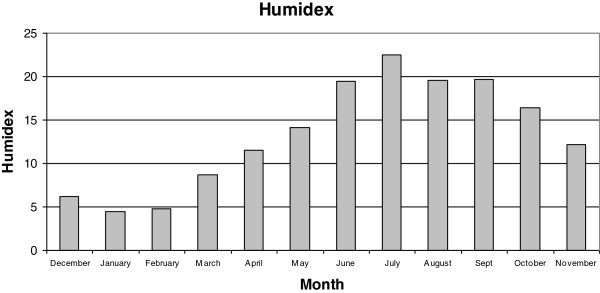
Monthly humidex in 2008.

**Figure 5:**
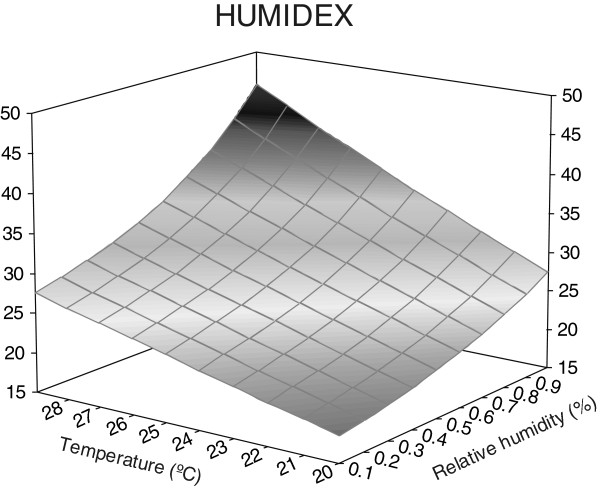
Humidex as a function of temperature and relative humidity.

**Figure 6:**
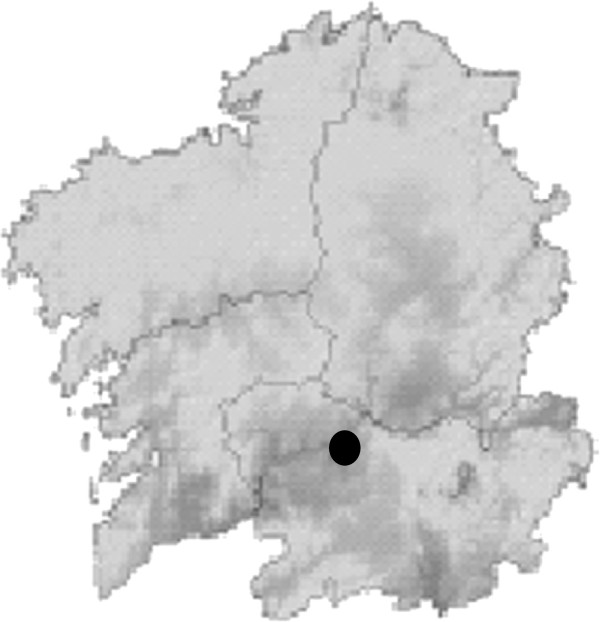
Temperature distribution in Galicia, in July.

**Figure 7:**
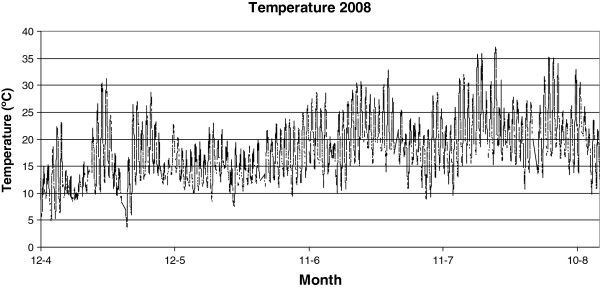
Instantaneous temperature in 2008, in Ourense.

**Figure 8:**
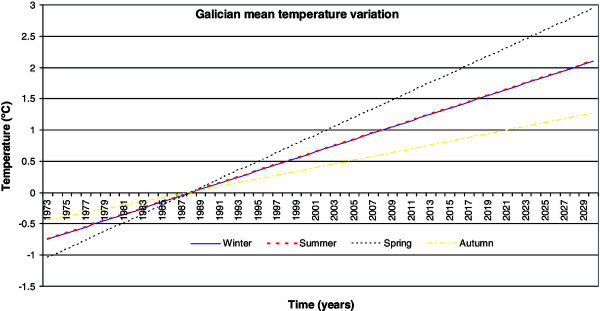
Yearly temperature increment.

**Figure 9:**
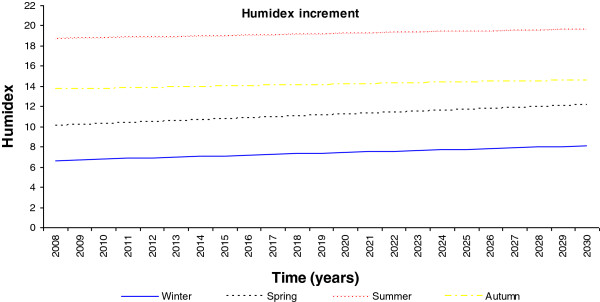
Yearly humidex increment.

**Figure 10:**
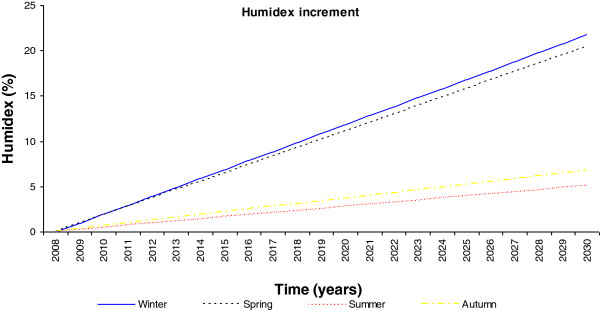
Yearly perceptual humidex increment.

**Figure 11:**
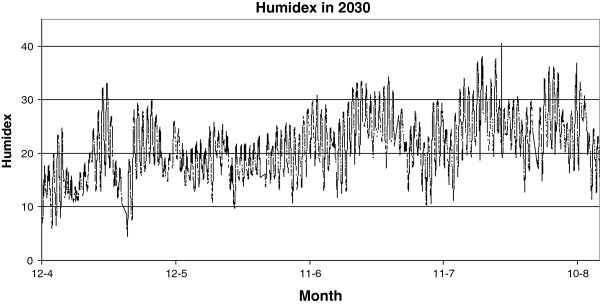
Instantaneous expected humidex in 2030.

In this paper, 50 weather stations were selected to sample the weather conditions in this humid region over the last 10 years. As mentioned earlier, Galicia, located in the northwest of Spain, experiences warm summers with mean temperatures of 20°C, and winters with 6°C, as seen in Figure 2. Despite these warm temperatures, a higher relative humidity of 70% is prevalent at all times, implying a mean humidex value of above 23 during July, as evident in Figures 3 and 4.

This effect is due to the increase in the humidex with temperature and relative humidity, as shown in Figure 5. This reveals that if we increase the relative humidity by 10% for the same value of outdoor temperature, the humidex shows the maximum value with maximum relative humidity. On the contrary, if we maintain the relative humidity and increase only the temperature it shows the maximum value with maximum relative temperature. Consequently, in such humid climates a clear tendency for higher humidex values is observed, particularly during the end of June.

Once this relationship is analyzed for the particular Galicia mean conditions, Galician temperature distribution should be analyzed in July for each region as in Figure 6, where the highest temperatures zones are shown in pitch dark color.

From a statistical study of the instantaneous values of the meteorological station, the region with the highest mean maximum temperature was identified. This region (see Figure 6) with a dark circle was found to be located near Ourense town and its instantaneous temperature values are shown in Figure 7. Here its maximum temperature during July and August, are shown, with values over 35°C. These values, however, are related to real weather heat stroke, and therefore, these regions begin to appreciate the new risk prevention strategies based on the humidex to cope with such a situation. Once the region with the higher health risk was identified, the effect of climatic change was analyzed. However, in recent studies, when Planet Global Heating was analyzed for the Galicia climate, it showed a rise in the average temperature each season with a slight drop in the summer wind frequency. Thus, the effect of climate change can involve a new situation with new potential regions of risk. Further, this is the right time to consider developing new methods to prevent this hazard influenced by city design, like buildings and roads materials [19].

To analyze the future climatic change effect, it is necessary to study the weather changes over the last 10 years. The results revealed that during the last 10 years, the winter season presents the better comfort conditions with a mean humidex of 7, while the summer season presents the highest humidex with a mean value of 18. Therefore, due to the higher relative humidity the whole year round, this humidex value reveals a clear dependence on the outdoor temperature.

From the data sampled and with the predicted change in the climatic conditions for these regions, new zones with the potential of heat stroke risk in the next 20 years can be identified. Thus, Global Planet Heating presents a clear picture of the outdoor comfort conditions reaching unbearable values. An increase in the mean Galician temperature for next 20 years has been predicted to be 1.35°C during the summer and winter seasons; however, the more extreme increment of 1.89°C will be expected during the spring season [3]. The lowest outdoor temperature increment is expected during the autumn season, with a rise of 0.81°C, as seen in Figure 8.

From these predicted temperature changes it is possible to estimate the seasonal humidex for the next 20 years, as shown in Figure 9. This reveals that during the summer season a maximum value of 21 will be reached and during the winter season a minimum mean value of 10 will be reached. Further, although it is known that the spring season will experience a higher temperature increment over the next few years, with respect to the existing conditions, the winter season will experience a slightly higher humidex increment of 1.54 with respect to the 1.10, 0.66 and 0.66 degree rises in the spring, summer and autumn seasons, respectively, as evident in Figure 10. This effect must be related to the higher relative humidity value of the region.

On analyzing the instantaneous effect of climate change in Ourense, the hottest point on the comfort conditions it is evident that maximum outdoor temperatures with values near 40°C are prevalent towards the end of July, as seen in Figure 11. Right through the summer the outdoor air relative humidity is around 90%, which is a typical value for this humid region. Therefore, the humidex reveals a clear increase in keeping with the temperature change. This increment reaches a maximum value of 41 related to distinct discomfort and health risk.

On analyzing the increase in temperature due to climate change and extrapolating for the instantaneous outdoor conditions of the year 2030, we obtained the data shown in Figure 11. This indicates that the humidex will experience an increase in its maximum value of 43, which is considered dangerous ambience.

These temperatures and relative humidity were sampled in the climatic stations located near the town, and therefore, these values will reveal an increase in the zones identified within the town. This is the “urban heat island effect” which causes individuals living in cities to be susceptible to a high degree of risk of death when the temperature and humidity are high as compared with those living in the suburban and rural areas [2]. Therefore, future research work is warranted to predict this temperature increment.

Future risk prevention in populations due to heat stroke must consider these predictions as a good index to improve the future planning and town design. For example, although thermal comfort is difficult to achieve passively, shading is one of the most significant properties that emerges with increased vegetation, thus mitigating the heat stress [20]. However, new software resources can guide architects to identify new solutions to treat outdoor spaces based on human thermal stress [21].

Finally, research work in the future is necessary to define a better calibration methodology [22] of the index to improve the correlation with empirical data, and consequently, generate a greater percentage of correct predictions. This calibration must factor in parameters like air pollution which will prevent the model from overestimating the mortality risk from unfavorable weather conditions [15].

## Conclusions

Galicia experiences warm summers with mean temperatures of 20°C, and winters with 6°C. Despite these warm temperatures, the higher relative humidity of 70% that prevails throughout the year indicates a high mean humidex value.

In this study, 50 weather stations were selected to sample the weather conditions in this humid region over the last 10 years to identify the zones experiencing the highest mean maximum temperature. However, Planet Global Heating was analyzed in the recent studies for Galician climate causing a rise in the average temperature that involves a new situation with new potential regions of risk.

From the data sampled and with the predicted changes in climatic conditions for these regions, the new regions with the potential of heat stroke risk in the next 20 years can be predicted. From these predicted temperature changes, the humidex for the next 20 years for each season can be estimated.

Specifically, an increase of 1.35°C in the mean Galician temperature for next 20 years during the summer and winter seasons was predicted; however, the more extreme increase of 1.89°C will be expected during the spring season. The lowest outdoor temperature increment is expected during the autumn season, with an increment of 0.81°C. Therefore, the humidex will show a mean increment from 7 to 10 and from 18 to 21, in the winter and summer seasons, respectively. On further analysis, the temperature increase due to climate change, extrapolating for the instantaneous outdoor conditions of the year 2030, makes it evident that this humidex will experience an increase in its maximum value of 43, which is related to dangerous ambience at the hottest point.

Finally, the humidex values are expected to reach the maximum supportable levels, and therefore, this fact must be taken into account in future town design. Further, future risk prevention in the population due to heat stroke must consider these predictions as an index to improve the future realizations. As an example of this methodology, the high risk regions will need to change their building construction materials and design characteristics to prevent risky situations, as well as to reduce their energy consumption.

## Competing interests

The authors declare that they have no competing interests.

## Authors’ contributions

JO Design of the study and humidex implementation. AC weather modelling and data analysis. ARF Design of the study and data analysis. GR Design of the study and climate change analysis. All authors read and approved the final manuscript.
